# An Energy Efficient Stable Election-Based Routing Algorithm for Wireless Sensor Networks

**DOI:** 10.3390/s131114301

**Published:** 2013-10-24

**Authors:** Jin Wang, Zhongqi Zhang, Feng Xia, Weiwei Yuan, Sungyoung Lee

**Affiliations:** 1 School of Computer and Software, Jiangsu Engineering Center of Network Monitoring, Nanjing University of Information Science & Technology, Nanjing 210044, China; E-Mails: wangjin@nuist.edu.cn (J.W.); jsczzzq@163.com (Z.Z.); 2 School of Software, Dalian University of Technology, Dalian 116620, China; E-Mail: f.xia@ieee.org; 3 Computer Engineering Department, Kyung Hee University, Suwon 449-701, Korea; E-Mail: weiwei@oslab.khu.ac.kr

**Keywords:** wireless sensor networks, mobile sink, clustering, multi-hop, lifetime

## Abstract

Sensor nodes usually have limited energy supply and they are impractical to recharge. How to balance traffic load in sensors in order to increase network lifetime is a very challenging research issue. Many clustering algorithms have been proposed recently for wireless sensor networks (WSNs). However, sensor networks with one fixed sink node often suffer from a hot spots problem since nodes near sinks have more traffic burden to forward during a multi-hop transmission process. The use of mobile sinks has been shown to be an effective technique to enhance network performance features such as latency, energy efficiency, network lifetime, *etc.* In this paper, a modified Stable Election Protocol (SEP), which employs a mobile sink, has been proposed for WSNs with non-uniform node distribution. The decision of selecting cluster heads by the sink is based on the minimization of the associated additional energy and residual energy at each node. Besides, the cluster head selects the shortest path to reach the sink between the direct approach and the indirect approach with the use of the nearest cluster head. Simulation results demonstrate that our algorithm has better performance than traditional routing algorithms, such as LEACH and SEP.

## 1. Introduction

Technology advancements in the areas of micro-mechatronic systems and wireless networks, *etc.* have allowed the rapid development of wireless micro-sensors for wireless communications. Wireless Sensor Networks (WSNs) comprise massive amounts of sensor nodes which make up the network for monitoring the region of interest and feed data about the targets or events of interest back to the end-users. WSNs usually include tiny, inexpensive and resource limited devices which communicate with each other in a multi-hop manner. WSNs can be widely used to perform military tracking and surveillance, natural disaster relief, hazardous environment exploration and health monitoring, *etc.* [1–3].

Due to the fact that it is impractical, if not impossible, to recharge sensor nodes, it is very important to design energy efficient routing algorithms or protocols to improve the energy efficiency of sensors by balancing and minimizing the energy consumption, and thus prolong network lifetime for WSNs [4]. In general, the sources of the energy consumption consist of three parts, namely sensing, processing and communication. We only consider the energy consumption during the communication process due to the fact that to transmit one bit of message consumes around 3,000 times more energy than to process the message.

An unbalanced energy assumption phenomenon occurs when the one-hop neighboring sensors deplete their battery power, and those sensors far away may still have more than 90% of their initial energy unused [5–7]. When comparing with sensors far from the sink, nearby sensors are shared by more sensor-to-sink paths, thus they have heavier message relay loads, and consume more energy [8]. To solve this problem, many energy efficient routing algorithms and protocols have been proposed in recent years, including clustering based routing protocols [9–16], mobile sink based routing protocols [17–22], power-aware routing [23–25] and multi-level transmission radii routing [26].

**Figure 1.**
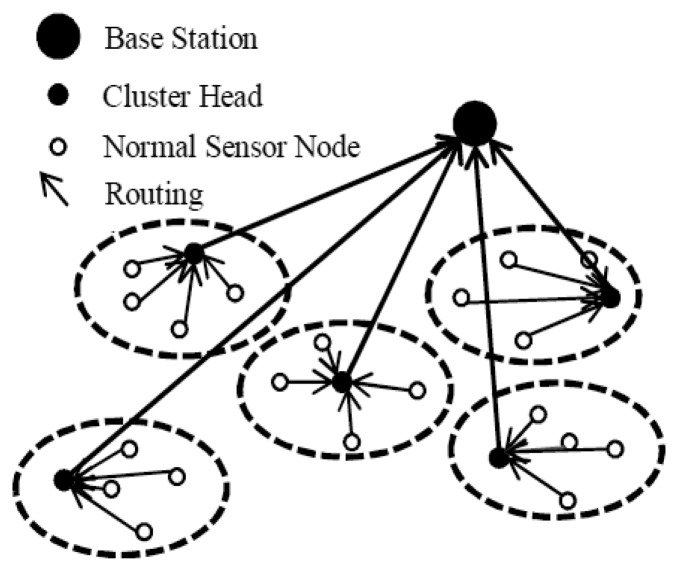
Clustering network topology.

Clustering has characteristics such as scalable, energy-efficient, lower latency, *etc.* which make it a popular technique for WSNs. The idea is to select a set of cluster heads from the set of nodes in the network, and then cluster the remaining nodes with these heads [12]. The data gathered are transmitted through cluster heads to remote base stations or sink nodes. However, sink nodes are always fixed, which could result in the neighboring nodes to dying much faster and causing network partition as well as isolated sensors. A typically clustered sensor network is illustrated in Figure 1.

The use of mobile sinks can potentially provide energy-efficient data collection with well-designed networking protocols for WSNs [27]. When using the mobile sink in practice, the sink nodes can be attached to vehicles, animals or people that can move inside the region of interest. Usually, static sink nodes are not very efficient [28]. Although single hop data collection is feasible in networks deployed in small regions, the multi-hop transmission manner is more commonly used in large sensor areas [29]. Intuitively, mobile sinks gain advantages by mitigating the so-called hot spot problem, balancing energy among sensor nodes, prolonging network lifetime, reducing transmission latency, and improving network performance by periodically accessing some isolated nodes into the network.

In this paper, we propose a Mobile sink-based improved algorithm for Stable Election (MSE) with non-uniform node distribution for WSNs. In this improved algorithm, the trajectory of mobile sinks locates in the centre line of the sensing field. Sink nodes move back and forth along the designed trajectory. The network is divided into several clusters based on the Stable Election Protocol (SEP) [30]. Each cluster head collects data and feeds it to the mobile sink.

The rest of the paper is organized as follows: Section 2 describes some related work, and our system model is provided in Section 3. In Section 4, our proposed MSE algorithm is explained in detail. Section 5 presents extensive simulation results and analysis. Section 6 gives a discussion of our work and finally Section 7 concludes this paper.

## 2. Related Work

Low-Energy Adaptive Clustering Hierarchy (LEACH) is a classic clustering algorithm for WSNs. It is a clustering-based protocol that utilizes randomized rotation of local cluster heads to evenly distribute the energy load among the sensors in the network. As the authors claimed, LEACH reduces communication energy by as much as 8 times compared with direct transmission and minimum-transmission-energy routing [9]. Its advantages can be summarized as follows: first, it can prolong the network lifetime compared to the original plane routing protocol and static clustering algorithms. Second, the cluster heads fuse the data collected from the corresponding areas, and transfer it to the sink node, which could effectively raise the energy use ratio. Finally, LEACH distributes the task among every sensor node, reducing the overload of individual nodes. LEACH-C was proposed in [10] to cope with the disadvantages of LEACH. It uses a central control algorithm to form clusters, which distributes cluster heads more evenly throughout the network. To make sure the energy load is evenly distributed among all nodes, the base station computes the average node energy. Nodes with energy below the average cannot be used as cluster heads for the current round [10].

However, LEACH-type protocols have some disadvantages. First, the algorithm offers no guarantee about the placement and number of cluster head nodes. Second, if the cluster head dies in round *n*, the whole cluster is unable to transfer its data to the base station until the next round. This intermittent failure of clusters could be a disaster when monitoring a region in real-time. Third, the individual sensor nodes transfer their data to the cluster head through single-hops, which is not suitable for large-scale networks. Therefore, further research has been undertaken into some of these issues.

The main idea in Power-Efficient GAthering in Sensor Information System (PEGASIS) is to make the energy load distribution more even among sensors for WSNs. Each node will receive from and transmit to close neighbors and take turns being the leader for transmissions to the base station [11]. It assumes that all nodes have global knowledge of the network; the base station is fixed at a far distance from the sensor nodes; the sensor nodes are homogeneous and energy constrained with uniform energy; and the energy cost for transmitting a packet depends on the distance of transmission. PEGASIS builds a chain to ensure that all nodes have close neighbors. When a node dies, the chain is reconstructed to bypass the dead node.

The Hybrid Energy-Efficient Distributed (HEED) [12] algorithm selects cluster heads according to a hybrid of node residual energy and a secondary parameter, such as node proximity or node degree. There is a tradeoff between extending the time until the first node dies (FND) and the time until the last node dies (LND). In [13], an evolutionary-based routing protocol has been proposed to obtain a better compromise between stability and network lifetime. It can guarantee a better tradeoff between the lifespan and the stability period of the network with efficient energy utilization. As the tradeoff that exists between network lifetimes and sensing coverage is the major problem in fixed sink networks, the authors in [31] proposed an energy-aware coverage-preserving hierarchical (ECHR) algorithm which accommodates energy-balance and coverage-preservation.

In [14], a distance aware intelligent clustering (DAIC) was proposed. The key concept is dividing the network into tiers and selecting the high energy CHs at the nearest distance from the base station. In [15], an Energy-Efficient Unequal Clustering (EEUC) mechanism for periodical data gathering in WSNs is proposed to address the hot spots problem. It partitions the nodes into clusters of unequal size, and clusters closer to the base station have smaller sizes than those farther away from the base station. In [16], an energy-aware clustering algorithm (EADC) was proposed using competition range to construct clusters of even sizes. The routing algorithm of EADC increases forwarding tasks of the nodes in scarcely covered areas by forcing cluster heads to choose nodes with higher energy.

Recently, several applications which introduce sink mobility into the wireless sensor networks have appeared. In some applications, mobile elements have been taken forward to attach network node for data collection [32–35]. It is very promising to use mobile sink to improve network lifetime without causing negative impacts to the network. This is because the role of hot spot node will rotate among most sensors, which will basically balance the traffic load throughout the whole sensor network.

In [17], a mobility-based clustering (MBC) protocol for WSNs with mobile nodes is proposed. The authors consider residual energy together with the current speed of each sensor node. A threshold value is multiplied by the factors representing the residual energy and the current speed of a node. Using this threshold, the nodes with more residual energy and lower speed may have more probability to be selected as cluster heads. MBC used a heuristic mechanism in which each sensor wakes itself up one timeslot before its scheduled timeslot according to the TDMA schedule and goes back to sleep mode after its timeslot.

A network infrastructure based on the use of controllably mobile elements was discussed in [36], with the essential of reducing the communication energy consumption at the energy constrained nodes and, thus, increasing useful network lifetime. The controllable mobile infrastructure can reduce energy consumption at the energy constrained nodes and, thus, increase useful network lifetime. In particular, the infrastructure focuses on network protocols and motion control strategies. The significant issue to be noticed is that the controllably mobile infrastructure tests using a practical system and do not assume idealistic radio range models or operation in unobstructed environments.

A novel geographic routing for mobile sinks has been proposed by [37] to address the issue that frequent location updates of mobile sinks may lead to both rapid energy consumption of the sensor nodes and increased collisions in wireless transmissions. The proposed scheme takes advantage of the wireless broadcast transmission nature of wireless sensor nodes. When a sink moves, the new location information is propagated along the reverse geographic routing path to the source during data delivery.

An Energy-Balanced Data Collection (EBDC) mechanism using a mobile data collector in WSNs was proposed in [38]. EBDC considers a circular monitoring region which has been geographically partitioned into a number of circular tracks. To cope with the energy unbalance problem, the authors determined the trajectory of a mobile data collector (or mobile sink) such that the data-relaying workloads of all sensors can be totally balanced. In [39], based on sensor transmission range *r* and the velocity of the mobile sink *v*, the authors evaluated the energy performance for both static and mobile sinks. They evaluated their algorithm in C++ simulator with limited energy. The authors found an balance for the mobile sink's moving speed, making sensors meet mobile sink more commonly, besides a sufficiently long session interval for the sensor and sink to successfully exchange one potentially long packet.

The Backbone-based Virtual Infrastructure (BVI) approach has been proposed to avoid the routing structure construction [18]. The BVI approach supports sink mobility without global position information. However, BVI networks are always considered as single hop networks, which makes the tree organize into too many cluster heads. Thus, in [19], a novel BVI-based communication protocol to support sink mobility without global position information was proposed. The authors used multi-hop clusters and rendezvous cluster heads to reduce the number of cluster heads.

In [20], the authors propose a novel localized Integrated Location Service and Routing (ILSR) scheme for data communications from sensors to a mobile sink in wireless sensor networks. In ILSR, sinks update their location to neighboring sensors after or before a link breaks and whenever a link creation is observed. ILSR is the first localized protocol that considers both unpredictable and controllable sink mobility. It further reduces message cost, without jeopardizing this property, by dynamically controlling the level of location updating.

In [21], to address the issue that a mobile sink with constant speed has limited communication time to collect data from sensor nodes deployed randomly, a Maximum Amount Shortest Path (MASP) collection scheme has been proposed. The MASP scheme can increase network throughput and conserves energy by optimizing the assignment of sensor nodes. In [22], a simulation-based analysis of the energy efficiency of WSNs with static and mobile sinks was proposed. It focused on mobility path of the sink and duty cycling value of the nodes. It also revealed that it is important to consider both *E*_max_ and *E*_bar_ in the energy analysis of a routing protocol, as improvement in one can result in degradation of the other and *vice versa*. It has also been observed that adopting a mobile sink and reducing the duty cycle of the nodes does not necessarily reduce the energy dissipation of the WSNs.

In [40], the authors proposed an improved stable election protocol based on mobile sinks for WSNs. However, the only thing that was taken into consideration was to combine SEP with the mobile sink which makes the evaluation of network lifetime not long enough. In this paper, we try to improve upon the work in [40], and further improvements with more explanation and comparisons will be proposed in the following sections.

## 3. System Model

### 3.1. Basic Assumptions

We make the following basic assumptions for WSNs in this paper:

(1) All sensor nodes are fixed after deployment;
(2) Each sensor node has a unique ID;
(3) Links are symmetric;
(4) There are no obstacle objects between communication pair;
(5) Sensor nodes are location-aware and can adjust their transmission power based on distance.

As can be seen from the assumptions above, the network is not assumed to be homogenous. It can be heterogeneous with various types of sensors and sink nodes (static or mobile ones). Some advanced sensor nodes and mobile sink nodes with more powerful energy supply can be applied here.

### 3.2. Network Model

**Figure 2.**
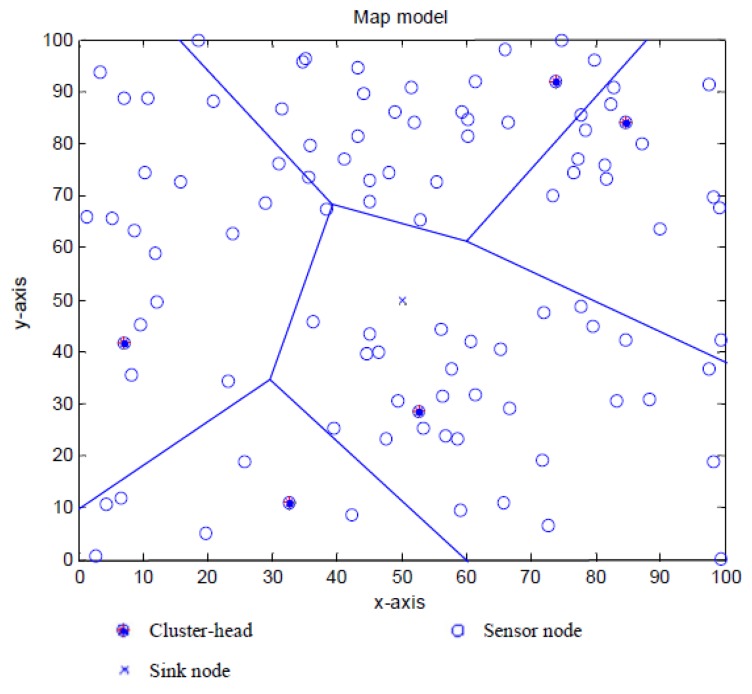
A non-uniform distributed WSN.

A non-uniform distributed WSN is shown in Figure 2, where a static sink locates at the center of the network. In this paper, we consider a network with N sensors randomly dispersed in a rectangular network.

The network is clustered into a group of clusters. Sensors are selected as cluster heads based on *T*(*n*), which will be discussed in Section 4. The sensors will transmit their sensed data to the sink through cluster heads via a single-hop or multi-hop transmission.

The network model can be described as an undirected connectivity graph *G*(*S*,*E*), where *s* is the set of all sensor nodes and *E*(*i*,*j*) is the set of wireless link between node *i* and node *j*. To indicate a sensor node condition, a function with position, residual energy, initial energy, and communication range is considered in Equation (1) where (*x*(*i*,*n*), *y*(*i*,*n*)), represents the position, *e*(*i*,*n*) is the residual energy, *E*(*i*,*n*) is the initial energy, and *R_i_* is the transmission range:

(1) $$\Psi_{i}(n)=f((x(i,n),y(i,n)),e(i,n),E(i,n),R_{i})$$

**Figure 3.**
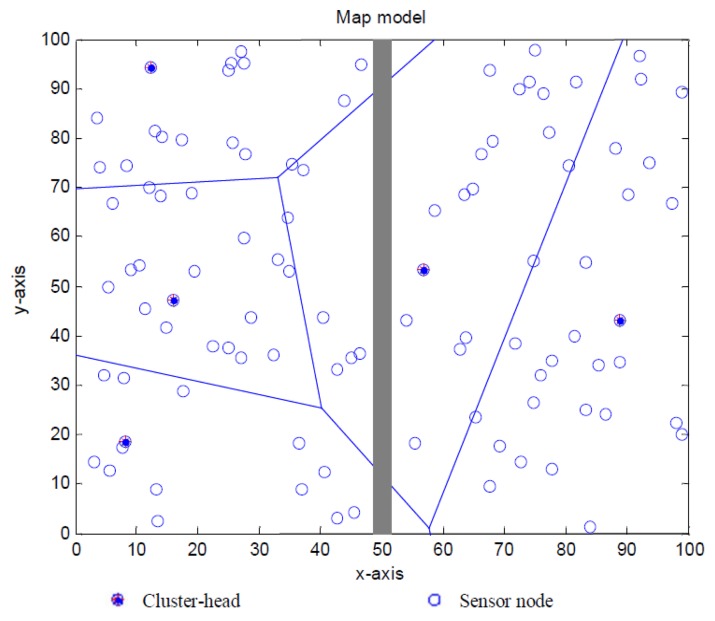
A non-uniform distributed WSN with a mobile sink moving on the centre line.

In terms of energy consumption and network lifetime, a mobile sink based strategy is proposed in Figure 3 to achieve better network performance, where the gray thick line is the predetermined movement path for mobile sink. As is shown in Figure 3, every circle indicates a sensor with position of (*x*(*i*,*n*), *y*(*i*,*n*)) and residual energy of *e*(*i*,*n*). Mobile sink node will move back and forth along the path in order to gather data packets from cluster heads.

### 3.3. Energy Model

We use the first order radio energy model [10] here in Equation (2). To transmit an *l*-bit length message through a distance *d*, the energy consumption by the radio is given by:

(2) $$\begin{array}{c} E_{Tx}(l,d)=E_{Tx-\text{elec}}(l)+E_{Tx-\text{amp}}(l,d)\\ \;=\{\begin{array}{c} lE_{\text{elec}}+l\varepsilon_{fs}d^{2},d<d_{o}\\ lE_{\text{elec}}+l\varepsilon_{mp}d^{4},d\geq d_{o} \end{array} \end{array}$$

where *E_Tx_*_−_*_elec_* represents transmitter electronics, *E_Tx_*_−_*_amp_* represents receiver electronics, *E_elec_* is the energy expended to transmit or receive one bit data, *ε_fs_* and *ε_mp_* illustrate the amplifier model we use, and *l* is the length for data waiting to be transmitted. The electronics energy *E_elec_* depends on factors like the digital coding, modulation, filtering, and spreading of the signal, whereas the amplifier energy varies according to the distance *d* between a receiver and a sender. When, *d* < *d*_0_, a free space channel model is accepted, while multi-path channel model is used when *d* ≥ *d*_0_.

We have $d_{0}=\sqrt{\varepsilon_{fs}/\varepsilon_{mp}}$ by equaling *ε_fs_d*^2^ and *ε_mp_d*^4^, *ε_fs_* where represents free space fading and *ε_mp_* represents multipath fading. To receive the message, the radio consumes:

(3) $$E_{Rx}(l)=E_{Rx-\text{elec}}(l)=lE_{\text{elec}}$$

The total energy consumption in the network is calculated in Equation (4), where *E_DA_* represents the energy for data aggregation and *N* is the number of nodes distributed uniformly in the network:

(4) $$E_{\text{tot}}=L\cdot \left(\begin{array}{l} 2NE_{\text{elec}}+NE_{DA}\\ +\varepsilon_{mp}k\cdot d_{\text{toBS}}^{4}+N\varepsilon_{fs}\frac{M^{2}}{2\cdot \pi \cdot k} \end{array}\right)$$

The optimum number of clusters can be found by setting the derivative of *E_tot_* with respect to k to zero, which is shown in Equation (5) as follows:

(5) $$k_{\text{opt}}=\sqrt{\frac{N}{2\pi }}\sqrt{\frac{\varepsilon_{fs}}{\varepsilon_{mp}}}\frac{M}{d_{\text{toBS}}^{2}}$$

(6) $$P_{\text{opt}}=\frac{k_{\text{opt}}}{N}$$

## 4. Our Proposed MSE Algorithm

In this paper, a modified Stable Election Protocol (SEP) which employs a mobile sink, with nonuniform node distribution for the WSNs, is proposed. The selection of cluster heads is based on the minimization of the associated additional energy and residual energy in each node. Additionally, the cluster head selects the shortest path to reach the sink between the direct approach and indirect approach with the use of the nearest cluster head.

### 4.1. Route Set-up Phase

#### 4.1.1. Cluster Heads Selection

Same as [9], the threshold of cluster heads is set in Equation (7) as follows:

(7) $$T(n)=\left\{ \begin{array}{ll} \frac{P}{1-P[r\mathrm{mod}(1/P)]} & if\;n\in G,\\ 0 & \text{otherwise}, \end{array}\right.$$

where *P* is a ratio of cluster heads among all sensors, 1/*P* is the expected number of nodes in one cluster, *r* is the index of the current round and *G* is the set of nodes that have not been cluster heads in the last *r* mod(*1*/*P*) rounds.

In each round, sensor node generates a random number between 0 and 1. If the random number is smaller than the current *T*(*n*), it will be selected as a cluster head. After the sensor node is selected as a cluster head, its corresponding *T*(*n*) will be set to be 0. Hence, every random number between 0 and 1 will not be smaller than the corresponding *T*(*n*), which ensures that the cluster heads will not be selected twice within 1/*P* round. Sensor nodes which have not been selected as cluster heads will continue the selection with threshold *T*(*n*) which will increase as round increases. After the (1/*P*−1) round,*T*(*n*)=1. Thus, the remaining nodes which have not yet been cluster heads will be cluster heads in the last round.

In our proposed algorithm, we consider the network to be heterogeneous, where there are *m* percentage advanced nodes which have the additional energy factor (*α*) in itself compared with normal nodes. In [30], to deal with this kind of heterogeneous sensor network, SEP has been proposed, and discussed in detail. With these advanced and normal nodes, this kind of heterogeneous layout has no effect on the density of the network. Hence, the previous set of *P_opt_* has no need to change. We assume the initial energy to be *E*_0_. The energy of advanced node in our proposed sensor network is *E*_0_ ·(1+ *α*). The total energy of new heterogeneous network is calculated in Equation (8):

(8) $$N\cdot (1-m)E_{0}+N\cdot m\cdot E_{0}(1+\alpha )=N\cdot E_{0}\cdot (1+\alpha m)$$

Hence, the total energy increases by (1+ *α*·*m*) times. Virtually there are *n*·(1+ *α*·*m*) nodes with energy equal to the initial energy of a normal node. Based on equations of probabilities for advanced and normal nodes, which discussed in detail in [30], we improved the selection method with the residual energy of certain sensor nodes. As is shown in Equation (9), the weighed probability for normal nodes is:

(9) $$P_{\text{nrm}}=\frac{P_{\text{opt}}}{1+\alpha \cdot m}\cdot \frac{E_{\text{residual}}}{E_{0}}$$

where *P_opt_* is the optical percentage of cluster head, *α* is the factor of additional energy, *m* is the percentage of advanced nodes, *E_residual_* is the energy left in sensor nodes after certain rounds, and *E*_0_ is the initial energy of any nodes. Similarly, in Equation (10), weighed probability for advanced nodes is:

(10) $$P_{\text{adv}}=\frac{P_{\text{opt}}}{1+\alpha \cdot m}\times (1+\alpha )\cdot \frac{E_{\text{residual}}}{E_{0}}$$

SEP replaces *P_opt_* by the weighted probabilities discussed above. Define *T*(*s_nrm_*) as threshold for normal nodes and *T*(*s_adv_*) as threshold for advanced nodes. As is illustrated in Equation (11), for normal nodes:

(11) $$T(s_{\text{nrm}})=\{\begin{array}{ll} \frac{P_{\text{nrm}}}{1-P_{\text{nrm}}[r\mathrm{mod}(1/P_{\text{nrm}})]} & if\;n\in G'\\ 0 & \text{otherwise} \end{array}$$

where *r* is the current round, G′ is the set of nodes which have not become cluster heads within the last 1/*P_nrm_* rounds, and *T*(*s_nrm_*) is the threshold applied to a population of normal nodes. Similarly in Equation (12), for advanced nodes, we define:

(12) $$T(s_{\text{adv}})=\{\begin{array}{ll} \frac{P_{\text{adv}}}{1-P_{\text{adv}}[r\mathrm{mod}(1/P_{\text{adv}})]} & \text{if}\;n\in G^{'}\\ 0 & \text{otherwise} \end{array}$$

where *r* is the current round, G″ is the set of nodes that have not become cluster heads within the last 1/*P_adv_* rounds, and *T*(*s_adv_*) is the threshold applied to a population of normal nodes [30].

#### 4.1.2. Cluster Formation

**Figure 4.**
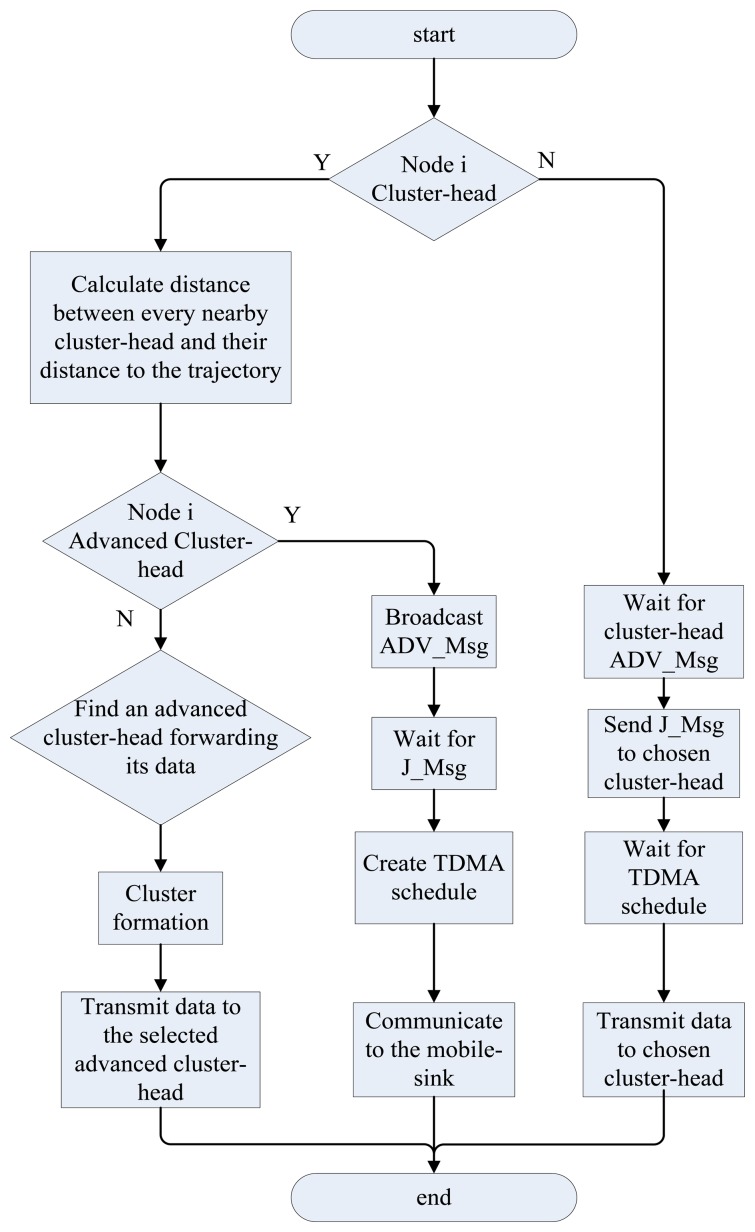
Flowchart of our proposed MSE method.

In this section, we construct a routing tree based on cluster heads set which have been elected and the communication procedures are illustrated in Figure 4.

During the broadcasting phase, each cluster head broadcasts an advertisement message (ADV_Msg) and its ID, location and type to sensors within its range using carrier-sense multiple access mechanism. Each normal cluster head will record the ID and location of an advanced cluster head with the strongest received signal strength (RSS).

During the decision phase, each non-cluster head node determines its cluster in this round by choosing the appropriate cluster head which has the strongest RSS of the ADV_Msg. After such a node has decided which cluster it belongs to, a join message (J_Msg) will be send to the corresponding cluster head with its ID and the chosen cluster head's ID. After the cluster head receives all the J_Msg, it sets up a TDMA schedule and transmits this schedule to the sensor nodes in its cluster. By using a TDMA schedule, collisions during messages transmission can be effectively avoided, and sensor nodes can be turned off if not on duty. This can effectively reduce the energy consumption for sensor nodes and prolong the lifetime of network.

**Figure 5.**
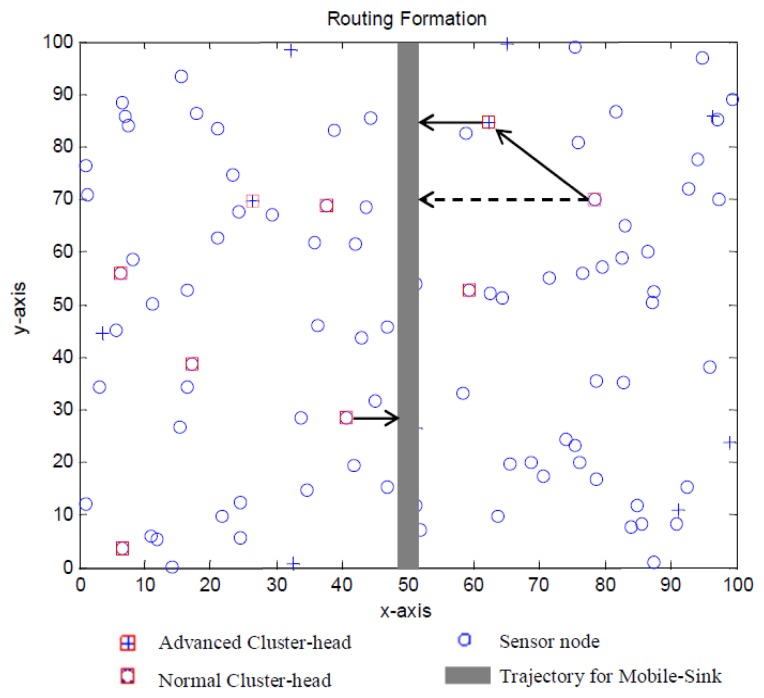
Routing strategy for MSE.

Assume a normal node's distance to the mobile trajectory and to the nearest advanced node, which has a distance of *d_m_* to the trajectory, are *d_n_* and *d_a_* respectively. As is shown in Figure 5, if the vertical distance from a normal cluster head to the mobile sink trajectory is longer than the distance between itself to its nearest advanced cluster head, it will calculate the vertical distance from the advanced cluster head to the trajectory. Finally, only when $d_{m}^{2}>d_{a}^{2}+d_{n}^{2}$, the normal cluster head will transmit its packet to the advanced cluster head. The packet will be fused and forwarded along with the data gathered in the cluster. As the distance will be calculated in each round, our algorithm's complexity equals to *O*(*n*^2^).

### 4.2. Route Steady Phase

In our proposed MSE algorithm, data of the interested region are sensed by the non-cluster-nodes in the network, and are transmitted to the respective cluster heads. To minimize the energy consumption in the network, the huge amount of data gathered in the cluster head ought to be fused into a single data message before transmitting to the mobile sink. After all the data in the cluster are gathered, the cluster head sleeps to further reduce energy consumption.

However, if there are two cluster heads with the same coordinate in the Y-axis, some collision would happen. To avoid this collision, we define the mobile sink moving once back and forth through the trajectory to be a round. In the first half round, the mobile sink only receives the data from the cluster heads in the left side of its trajectory, and the right part in the second half round.

### 4.3. Route Maintenance Phase

**Figure 6.**
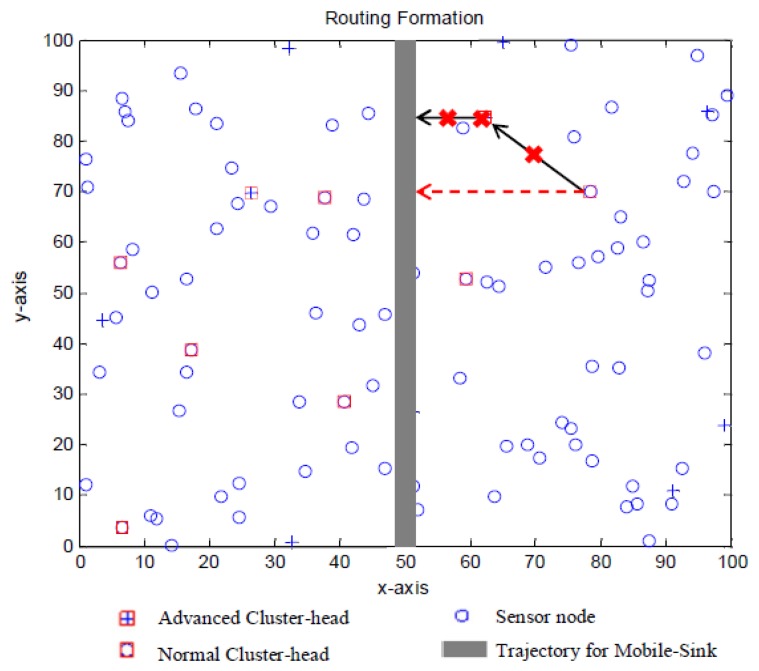
Maintain the routing path.

In real world implementation, as shown in Figure 6, there is a chance that the advanced cluster head will die in a certain round or somehow there is a block between some sensor nodes, causing unexpected failures. Once the advanced cluster head dies or is blocked, its corresponding normal cluster head will no longer have a next-hop, leading a certain area to be unreachable, and finally making the data inaccurate. However, reclustering a whole network only to solve one failure may result in significant waste of resources.

**Figure 7.**
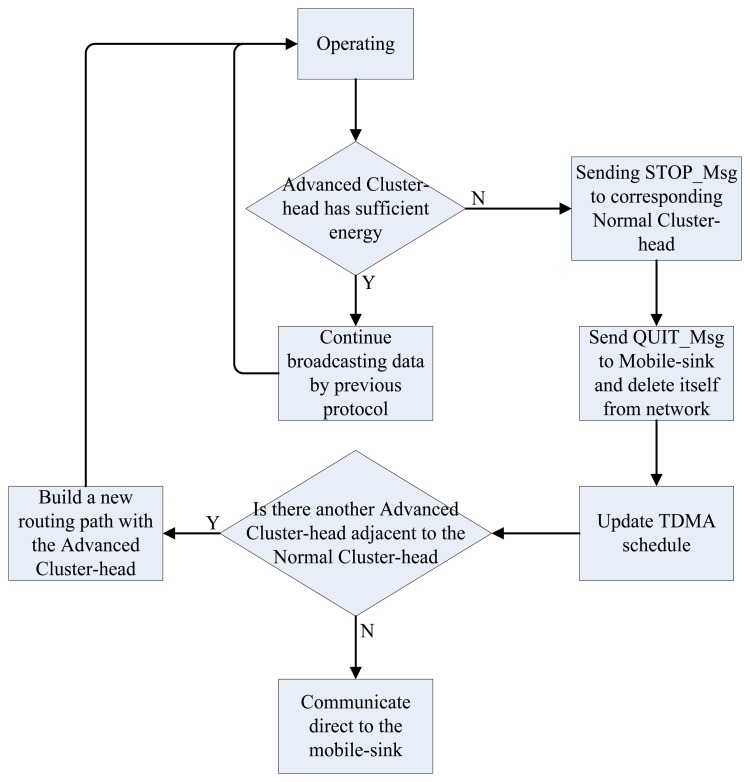
Flowchart for our maintenance method.

To solve this phenomenon, calculating the residue energy of any advanced cluster head is recommended. As shown in Figure 7, once the residue energy is not sufficient for the next data transmission and forwarding, it will send a STOP_Msg to its corresponding normal cluster head and delete itself from the sensor network. In the meantime, the TDMA schedule ought to be updated. After the normal cluster head receives the STOP_Msg, it will compare its distance to the adjacent advanced cluster head and trajectory. From this step on, the procedure is exactly the same as the set-up phase. As illustrated in Figure 6, once the original advanced cluster head dies, the corresponding normal cluster head carries out the procedure above immediately, and forwards its data direct to the mobile sink.

## 5. Performance Evaluation and Discussion

### 5.1. Simulation Environment

We use the MATLAB simulator to evaluate the performance of our proposed MSE algorithm. Simulation parameters are listed in Table 1, where 100 sensor nodes are distributed randomly in a rectangular region of 200 × 200 m^2^. There are 20% of advanced nodes which are equipped with 400% more energy than normal nodes (which means m = 0.2 and *α* = 4). Obviously, the network with high density of advanced nodes will have a relatively long lifetime.

### 5.2. Performance Evaluation

**Figure 8.**
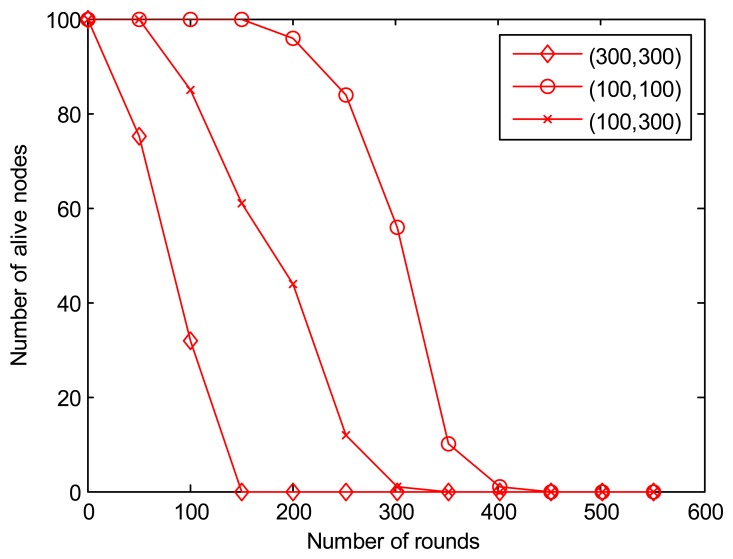
Influence on sink node locations.

Figure 8 shows that the energy consumption increases when it gets further away from the sink node, which shortens the network lifetime. The MSE algorithm consumes less energy with the mobile sink inside the network. However, we cannot explain why the decreasing rate of live nodes get slower in round 150–200 while the fixed sink lies in location (100, 300). To our best knowledge, this phenomenon may be caused by the uniform distribution.

**Figure 9.**
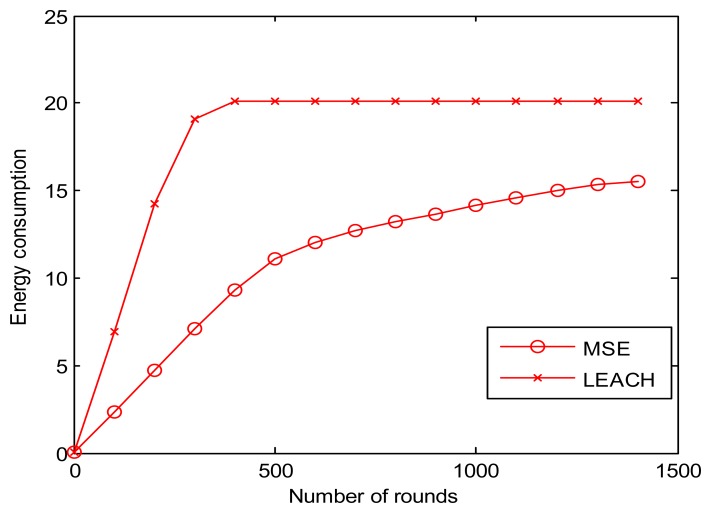
Energy consumption comparison.

Figure 9 shows that the energy consumption rate of LEACH is much larger than our MSE. Consequently, the energy in a LEACH network get drained much earlier than in a MSE one at the location about 400 rounds away from the sink node. The MSE energy consumption for both methods are almost linear before 500 rounds, while the linear part for the energy consumption curve of LEACH is before round 150. For the better illustration of these two energy consumption curves, we only choose the data within 1,500 rounds in order to magnify the bi-linearity of the energy consumption curve of LEACH.

**Figure 10.**
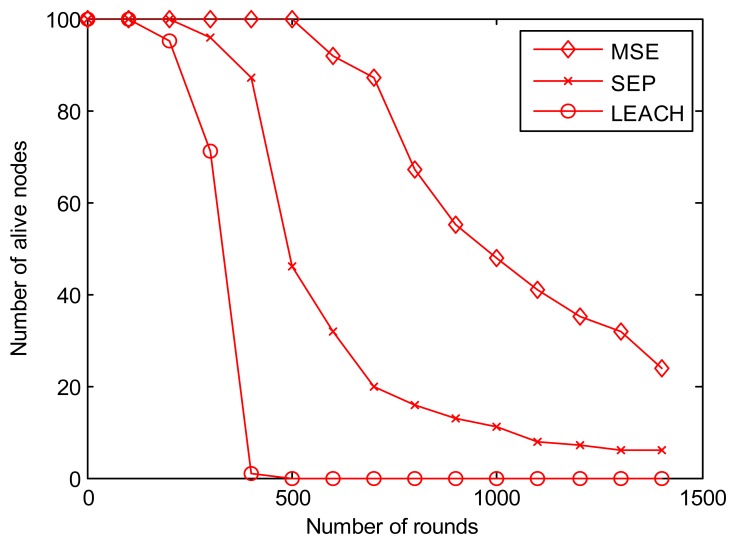
Network lifetime comparison.

Figure 10 shows the number of live nodes during the simulation lifetime. We choose 1,500 rounds here to have clearer view. We can find that the time when the first node dies in MSE is much longer than that in LEACH and SEP, nearly 2.5 times longer than SEP and 5 times longer than in LEACH. Figure 10 also indicates that the number of live nodes decreases more and much faster as time goes by.

Table 2 shows the round when the first node dies in the three protocols respectively. The longer the time for the first node to die, the more balanced the network will be. In a network which requires a more stable working time, the proposed MSE method will be more suitable.

**Figure 11.**
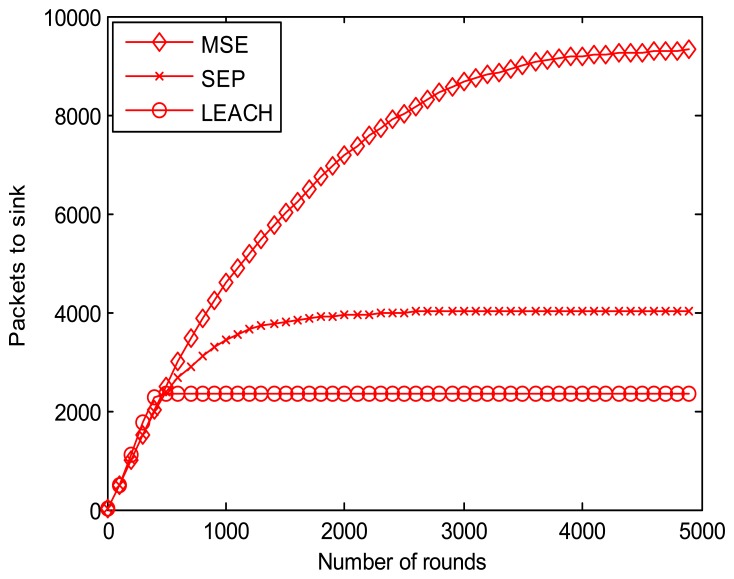
Packets received by the sink node.

Figure 11 shows the number of packets received by the sink. As illustrated, the result shows that MSE has a higher number of data received than LEACH and SEP. In the first 200 rounds, the three proposed algorithms have nearly the same packet delivery numbers. However, after 500 rounds, the sensors in the network with the LEACH protocol drain out totally and transmit no packets, while SEP and MSE continue delivering and forwarding data. The network using SEP drains out at about 2,500 rounds, while MSE lasts for almost 5,000 rounds. The amount of packets received by the sink node in the network using the MSE protocol is thus about 4 times larger than that of LEACH and 2 times larger than SEP.

## 6. Discussion

### 6.1. Mobile Sink Advantage

In fixed sink node networks, as sinks are always away from sources, the transmission paths from areas of interest to a sink node often form certain multihop routing paths. However, in this kind of routing path, the sensors close to the sink exhaust their energy very fast. In the calculation, sensors nodes located the furtherest from the sink have 90% residual energy when the one-hop neighbor nodes drain their energy out. Finally, this uneven energy consumption will lead to energy holes, area isolation, high transmission latency, and data inaccuracy.

With the aim to improve network performance, recent research has exploited mobile sinks. By introducing mobile sinks into sensor networks, optimization of energy efficiency, lifetime, and peer-to-peer delay can be achieved. Besides, with implemented mobile sinks, network isolation can be effectively mitigated. To realize mobile sinks in real-world implementation, special devices like gateways can be attached on taxis, animals, and humans. In this paper, one mobile sink with a trajectory along the central line of a rectangular region is proposed.

### 6.2. Trajectory

In real-world implementation, there is a chance that a mobile sink cannot move along the original trajectory due to some blocks ahead. In this scenario, a suboptimal trajectory should be established immediately. For instance, if a barrier blocks part of the trajectory and holds-up the mobile sink from moving on, the main idea of our solution is to make a cross-over. Once the mobile sink discovers the block ahead, it will soon scan from left to right and choose a direction with no barrier. A mobile sink moves on with previously stated method, however in every round the chosen direction should with the priority of moving back to the original trajectory. In the meantime, the location of the mobile sink should be updated to every sensor node.

### 6.3. Open Research Issues

Some basic assumptions were made when we discussed the system model, such as the fact the sensors were fixed after deployment, synchronized, symmetric, and location-aware. However, in real-world implementations, these assumptions are very difficult to realize. It will consume a lot of energy to synchronize and ensure the location awareness of the sensors. Hence, network lifetime cannot be as long as proposed in a theoretical environment. Besides, in a hazardous environment, such as a battle or regions which often attacked by typhoons, sensors may suffer from failures, displacement, and unsteadiness. Therefore, a new protocol with less assumptions and practical issue considerations may be needed.

## 7. Conclusions

In this paper, we described a SEP-based MSE method for energy efficient routing in WSNs. Our proposed MSE protocol forms hierarchical routing protocols by dividing the network into clusters and selecting cluster heads based on the fraction of advanced nodes with additional energy and the ratio between residual and initial energy. The MSE protocol shows promising performance in balancing the energy and prolonging network lifetimes. However, the trajectory in our proposed network is static, so when the node dies or the topology changes, the pre-located fixed trajectory may be unsuitable all the time. In the future, we plan to study networks with multiple mobile sinks and adjustable trajectories. Furthermore, a network with less assumptions should be constructed and the communication delay between sensor nodes should be taken into consideration too.

## Figures and Tables

**Table 1.** Simulation parameters.

**Simulation Parameters**	**Representation**	**Unit**
N	Number of sensor nodes	100
*E*_0_	Initial energy of sensor nodes	0.2 J
*E_DA_*	Data aggregation	5 nJ/bit/signal
*E_elec_*	Energy dissipation to run the radio device	50 nJ/bit
*ε_fs_*	Free space model of transmitter amplifier	10 pJ/bit/m^2^
*ε_mp_*	Multi-path model of transmitter amplifier	0.0013 pJ/bit/m^2^
*l*	Packet length	4,000 bits
*d*_0_	Distance threshold	${\sqrt{\varepsilon_{fs}/\varepsilon_{mp}}}_{m}$

**Table 2.** Round when first node dies.

**Algorithm**	**First Death Round**
LEACH	100
SEP	211
MSE	525
